# Supplementary material for: AVATREE: An open-source computational modelling framework modelling Anatomically Valid Airway TREE conformations
Source: PLoS One. 2020 Apr 3;15(4):e0230259. doi: 10.1371/journal.pone.0230259 (PMC7122715; doi:10.1371/journal.pone.0230259)
Supplement: Attachment — Submitted filename: AVATREE comment.docx [file pone.0230259.s001.docx]

I have read PONE-D-19-24923 which develops a computational modelling framework called “AVATREE” to define the personalized boundary conditions required for the simulation of pulmonary functions. Specifically, the AVATREE works as follows: (i) using image data to provide patient-specific representations of the structural models of the bronchial tree, (ii) establishing and extending 1D graph representations of the bronchial tree, (iii) generating 3D surface models of the extended bronchial tree models, (iv) producing probabilities visualization of airway generations on the personalized CT imaging data, and (v) offering an open-source toolbox in C++ and a graphical user interface integrating modelling functionalities. The AVATREE can successfully create anatomically valid airway tree conformations, which would be helpful to eventually predict gas flow and particle deposition characteristics in the healthy and diseased bronchial trees. In my view, this computational modelling framework is worth popularizing, and therefore I advise the paper to be accepted by the PLOS ONE.
